# Youth Knowledge of Physical Activity Health Benefits: A Brazilian Case Study

**DOI:** 10.1100/tsw.2006.283

**Published:** 2006-12-28

**Authors:** Susan Gail Zieff, Claudia Maria Guedes, James Wiley

**Affiliations:** Department of Kinesiology Public Research Institute San Francisco State University, San Francisco, CA, USA

**Keywords:** adolescent, physical activity, health knowledge, Brazil

## Abstract

This study presents the findings of a questionnaire-based investigation of knowledge about the relationship of physical activity to health among adolescent participants of a community-based physical activity intervention program in São Paulo, Brazil. Qualitative (inductive content analysis) and quantitative methods were applied to examine the participants responses to two open-ended questions concerning the health benefits of physical activity and the educational goals of the intervention. More than 75% of all participants stated that health benefits (of some type) are attained through participation in physical activity. More than 50% of participants reported that the goal of the intervention was to educate people about the importance of a healthy, active lifestyle. Adolescents understand the relationship of physical activity to health as reflected in their knowledge assessments; their lifestyle choices support these beliefs. These findings offer encouragement for the development and implementation of educationally oriented interventions aimed at providing physical activity information and programming.

## INTRODUCTION

Inadequate participation in health-related physical activity (PA) among adults and children has become a global phenomenon occurring in industrialized, developing, and third-world countries[1,2,3,4,5]. Decreasing rates of PA and increasing amounts of time spent in sedentary pursuits are also contributing to rapidly growing numbers of obese children and teenagers[6] who face potentially decreased life expectancy as adults[7,8]. Declines in PA participation typically begin in late childhood, accelerate during high school, and continue into the adult years, creating the context for ill health among adults[4,5]. Consequently, late childhood and adolescence are critical periods for the acquisition of both healthy and unhealthy behaviors[3]. High levels of childhood PA have been shown to predict for high levels of adult PA[5,9], as well as reduce the biological risk factors that are likely to impair health in adults[4,10].

### Physical Activity Knowledge and Behavior

Among the intervention programs designed to increase PA among children and adolescents, few offer an educational component linking behavior change to knowledge gain about specific ways to change and the important reasons for change. A growing body of evidence suggests that among adults, exposure to information about the health benefits of PA positively influences lifestyle changes[11]. Morrow et al.[12] suggest that PA knowledge would be more effective if individuals are able to identify specific PA behaviors considered useful for improving health. Yet, the high number of inactive adults also suggests that health information without advice about effective strategies for changing behavior is only minimally effective[12].

In one of few studies of the health information–behavior change relationship among adolescents, Simon et al.[13] demonstrated that after a 6-month intervention aimed at changing attitudes about PA, middle school students showed a significant improvement in PA patterns. However, before we can know if acquired information leads to behavior change in youth, we need to investigate if and how adolescents understand information emphasizing the role of PA in improving health. Many of the interventions in the literature have focused on elementary school children, with a resulting lack of information about the particular issues facing middle school students[14]. Few interventions report long-term behavioral change or contribute to our understanding of why the number of sedentary youth continues to increase, suggesting that such strategies need to be more effective[7,14].

The general objective of this study was to identify how youth understand and articulate health-related messages promoted by the PA intervention program, Agita Galera, the adolescent segment of the community-based, health program “Agita São Paulo” in the State of São Paulo, Brazil.

## METHODS

In 1996, the Physical Fitness Research Center from São Caetano do Sul (CELAFISCS) and the Office of the State Secretary of Health in São Paulo, Brazil established a multilevel program, “Agita São Paulo” (ASP) to provide information about the health benefits of PA and to coordinate related events and interventions for the more than 37 million inhabitants of the State of São Paulo[1]. ASP emphasizes the focused message “Physical Activity is Health: Accumulate 30 Minutes per Day,” following the then-published guidelines of the American College of Sports Medicine and the CDC (Centers for Disease Control and Prevention)[1,15].

Agita Galera (AG) is the youth segment of ASP. A “mega-event” for AG is held annually, with typical participation from over 6,500 schools and 6 million students, to raise awareness about the importance of an active lifestyle and to model the kinds of activities and events provided within local communities[1]. In addition, media announcements, advertisements, school lectures, and printed materials containing information about the health benefits of PA are provided to children attending schools participating in AG[16]. Through its design as a year-round program distributing educational items (videos, posters, stickers, games) to the involved schools, AG spreads its message of health-related PA[17].

The specific purpose of this research was twofold: (1) to determine how youth participants in a community-based, PA intervention program (AG) understand and communicate about the importance of PA in relation to health and (2) to determine if the participants understand that the message about the health benefits of 30 min of daily PA is the program’s educational objective.

A 22-item questionnaire was developed for internal consistency reliability and therefore included different questions measuring the same phenomenon[18]. The design of this tool included seven descriptive, closed-ended demographic questions (e.g., sex, age, school grade) and 15 open-ended questions. Seven of these open-ended questions asked about participants’ PA history and current behavior and eight questions investigated the participants’ conceptual understanding of the connections between PA and health (see Box). In addition, this project used a “grounded theory” design in which the researchers enter the research setting without a predetermined theory, instead allowing the data collected to determine the theory outcomes[18]. Open-ended questions provide the opportunity for participants to share their knowledge and experiences with few limitations imposed by the researchers. Both qualitative and quantitative data analysis methods were used to identify, discuss, and measure exemplary and illustrative responses to selected open-ended

**BOX 1**
**Selected Questionnaire Items about Physical Activity History and Behavior**

1. Is physical activity important? Why?
2. Do you engage in physical activity? How many times per week? How many minutes per day?
3. Do you practice physical activity alone or with others?
4. What type of physical activity do you practice?
5. Where do you practice your physical activity?
6. When you are physically active, for how many minutes?
7. How many minutes do you dedicate to practicing physical activity per week outside of school physical education?
8. What are the objectives of Agita Galera?

questions and to assess participants' current PA as active members of AG. These methods were chosen on the assumption that better results can be achieved through investigations that integrate frequency of responses and analysis of meaning in the process of interpreting data and developing conclusions.

This study presents the findings of a questionnaire-based investigation of knowledge about the relationship of PA to health among adolescents who participated in AG, a PA event held in 2004 in São Paulo, Brazil.

## Procedures

Twenty-three undergraduate research assistants and eight graduate research coordinators received 12 h of training in research techniques including participant recruitment, questionnaire delivery, and issues of professional responsibility. The San Francisco State University Institutional Review Board for the Protection of Human Subjects reviewed and approved the project. Informed consent forms were distributed to prospective research participants on-site and questions about the process were answered. Consent forms and completed questionnaires were coded to protect the identities of the participants.

The questionnaire was delivered on-site in Parque Chico Mendes, in Sao Caetano, a suburb of the city of Sao Paulo, during the AG mega-event on August 27, 2004 involving five schools. All youth participants in AG ages 14–18 were eligible to complete the questionnaire. The research assistants distributed themselves throughout Chico Mendes Park from 8 a.m. until 4 p.m. delivering the questionnaire to 255 of the approximately 300 participants (i.e., as many as possible and as time permitted), using random and snowball recruitment methods.

## Participants

Inclusion criteria for the participants were as follows: age 14–18 years and involved in AG. N = 55 received a consent form and were interviewed using the questionnaire. N = 22 were removed from analysis when they were found to not be involved in AG, leaving approximately 77% of the total number of participants for data analysis. The adolescent sample of participants in our study was predominantly male (59%), white (69%), and age 16 or under (82%).

These participants present a relatively high rate of PA (Table 1). The mean number of PA sessions is 3.7/week, and 60% of the sample reported three or more sessions per week, meeting and in many cases, exceeding, the minimum recommended by the CDC and ACSM[19]. The CDC and ACSM also recommend

**TABLE 1**
**Physical Activity Indicators**

| | N | % |
|---|---|---|
| 1. Number of sessions of PA per week | | |
| 0–1 | 18 | 7.8 |
| 2 | 73 | 31.6 |
| 3–5 | 88 | 38.1 |
| 6+ | 52 | 22.5 |
| Total | 231 | 100.0 |
| 2. Average number of minutes per week | | |
| 30 min or less | 25 | 10.8 |
| 31–60 | 77 | 33.3 |
| 61–120 | 90 | 39.0 |
| More than 120 min | 39 | 16.9 |
| Total | 231 | 100.0 |
| 3. Types of PA | N | % |
| Team sports | 136 | 58.9 |
| Fitness activities | 52 | 22.5 |
| Martial arts/dance | 34 | 14.7 |
| Other (e.g., skateboarding, ping pong, surfing) | 9 | 3.9 |
| Total | 231 | 100.0 |
| 4. Participation rates for AG | | |
| 1st time | 92 | 39.8 |
| 2nd time | 43 | 18.6 |
| 3rd time | 39 | 16.8 |
| 4th time | 26 | 11.2 |
| 5–11 times | 31 | 13.4 |
| Total | 231 | 100.0 |
| 5. Age in relation to first time in AG | | |
| 14 | 24 | 26.1 |
| 15 | 33 | 35.9 |
| 16 | 22 | 23.9 |
| 17–18 | 13 | 14.1 |
| Total | 92 | 100.0 |

a minimum of 30 min of PA per activity day, a standard reached by 89% of the participants. Over 60% of participants demonstrated an adherence to the AG program, having attended mega-events two or more times. The number of PA sessions and minutes per day were achieved through activities defined as "vigorous" rather than "lifestyle" or "moderate" PA[19,20] including team sports (e.g., soccer, volleyball, basketball), fitness (e.g., body toning classes, calisthenics, jogging), martial arts and dance (street dance, kung fu, capoeira), and other activities (e.g., ping pong, surfing, skateboarding).

## Data Analysis

Inductive Content Analysis (ICA) was used to analyze the selected open-ended questions (Table 2). ICA follows a multiphase process and allows for multiple responses[21]. First responses have value independent of other responses and are presented in the data analyzed here. However, first or more frequent responses do not necessarily hold the most important value[22].

**TABLE 2**
**Inductive Content Analysis Procedures**

| Process | Outcome |
|---|---|
| Translate and transcribe raw data | Identify frequent responses |
| Development of themes from transcribed responses | First-level themes |
| Independent assignment of first responses to themes by two researchers | Second-level themes |
| Consensus categories and first response assignments | Second-level themes confirmed, retained as third-level themes |
| Frequency distributions of third-level themes | Evaluate themes with highest frequency |
| Comparisons of theme, age, and gender frequency over subgroups | Evaluation of differences and their significance |

The process began with the two researchers, (both bilingual readers of Portuguese and English), translating and transcribing verbatim the complete response of each participant and entering these onto an Excel spreadsheet. Then, using the words of the participants (raw data), frequently appearing responses were identified and counted. From this preliminary analysis, first level themes were developed. “Theme” is the term used in ICA to indicate a grouping of responses that are interpreted and then coded to have similar meaning. Then, independently, the researchers coded the raw data for each response to the two selected open-ended questions using the first-level themes. These codes are labeled second-level themes. The level of intercoder reliability[23] at this stage of coding was quite high: kappa = 0.809, $p < 0.001$, for adjusted intercoder agreement in assigning first responses to the seven thematic categories associated with the open-ended question “Is Physical Activity Important? Why?”; kappa = 0.913, $p < 0.001$, for adjusted intercoder agreement in assigning first responses to the four thematic categories associates with the open-ended question “What are the objectives of Agita Galera?”

The researchers then rejoined to compare their assignment of second-level themes. Second-level themes that were agreed became third-level themes and preserved for continued analysis. Where different themes were coded for any specific response, the researchers re-examined the original text and negotiated about the interpretation of the data before performing the final coding of the themes. The themes are allowed to emerge directly from the text-based data, and represent it, as closely as possible. This procedure allows for the development of progressively inductive categories paralleling the reductive process of the text interpretation[21].

In the final stage of data interpretation, quantitative analyses of the coded themes were conducted. We examined frequency distributions of themes based on first responses and compared the relative frequency of themes in different subgroups of participants.

## RESULTS

The results of the ICA of the two selected open-ended questions are reported here: (1) “Is physical activity important? Why?” and (2) What are the objectives of AG? Typical responses for each subtheme are included below to demonstrate both the range of issues reported and to provide examples of participant responses.

From a review of the responses to the first question, seven themes emerged (Table 3A). Almost half of the participant first responses addressed specific health benefits of PA and included references to enhancement of the physiological system, the avoidance of disease, preservation of health, and improving mental health. "Health" and "Physical Development" were categorized separately to ensure that participants' responses pertaining to the improvement of specific elements of the physiological system were differentiated from the (performance) training benefits that come from engaging in systematic PA. Responses related to issues of beauty, body shape, and weight gain/loss were included in the category "Aesthetic Reasons". The theme "Quality of Life" referred to daily life benefits, while the category "Enjoyment" included comments about fun and meeting people through PA. Comments about the ways that PA delays or lessens the effects of aging were included in the theme "Prevent Aging".

**TABLE 3 A**
**Is Physical Activity Important? Why?**

| Theme | No. of First Responses | % of First Responses | Example Comments |
|---|---|---|---|
| Health | 99 | 42.8 | "It improves your circulation, respiration, and breathing." |
| Physical development | 56 | 24.2 | "To keep physically good for other activities." |
| Aesthetic reasons | 32 | 13.8 | "To help you stay in shape, to have a beautiful body." |
| Quality of life | 18 | 7.7 | "To enjoy life more." |
| Enjoyment | 16 | 6.9 | "It makes me feel good." |
| Don't know | 6 | 2.6 | "I do not know." |
| Prevent aging | 4 | 1.7 | "It prevents me being old." |

More than half of the participants stated that the aim of AG was to provide education and information about the role of PA in a healthy lifestyle in their first responses to the second question (Table 3B). Less than one-third of the participants viewed AG as primarily a vehicle for entertainment. The difference in "I don't know" responses between the two open-ended questions may indicate that participants are (importantly) receiving information about the health benefits from involvement in the program, but had difficulty expressing the program's specific goals. Five of the six participants responding "I don't know" to the question about the important of PA, were engaged in PA two to five times per week.

Female and younger participants were more likely to provide first responses related to educating for health and PA as the primary objective of AG (Table 3C). No other sex differences were found. In addition, the data demonstrates that type of activity selected does not influence the extent to which the objectives of the intervention are understood. This population engaged in 42 different fitness (e.g., weight training), martial arts (e.g., capoeira, street dance), and team sport activities (e.g., European handball), most of which could be characterized as "vigorous"[19,20]. Of the participants, 136 were involved in team sport. Eighty-nine (65.4%) of the team players reported "health/physical development" in their first response to the question: "Is physical activity important?"

Although the results of this study did not directly link acquisition of health knowledge about PA with changes in PA behavior, 60% of those engaged in PA three times per week, 45% of those involved in PA twice per week, and 38% of those performing daily PA reported "health" as their first response to the question "Is PA important? Why?" When the results of the four subthemes reflecting health-related issues were combined (health, physical development, prevent aging, and quality of life), 76% of first responses reported the healthful benefits of physical activity.

Adherence to the program was strong; 60% of the participants were returning for their second year or more of involvement in its events. Age of first involvement in AG was well distributed between ages 14 and 18, indicating that the educational materials and programming offered was suitable to youth of different ages. The quality of the text responses demonstrated the participants' emphasis on avoiding or reducing sedentary behavior. Many of their statements referenced their personal efforts to be more active and articulated their concerns about the health consequences of inactivity. Among these participants in AG, less attention was given to issues of beauty, body, and enjoyment than has been typically shown in the literature[24,25].

**TABLE 3B**
**What are the Objectives of Agita Galera?**

| Theme | No. of First Responses | % of First Responses | Example Comments |
|---|---|---|---|
| Educate for healthy/active lifestyle | 123 | 53.3 | "To bring the importance of PA to the people." |
| Entertainment | 68 | 29.4 | "To have fun and relax after school." |
| Don't know/like | 30 | 13.0 | "I don't know." |
| Avoid risk behavior | 10 | 4.3 | "To take people from the streets, from the drugs, and bring more health." |

**TABLE 3C**
**Agita Objectives by Gender and Age**

| | First Response for Agita Objectives | | | Total (%; N) |
|---|---|---|---|---|
| | Educate for Health/Active Lifestyle (%) | Entertainment (%) | Other (%) | |
| Gender | | | | |
| Females | 66.3 | 23.1 | 10.5 | 100.0; 95 |
| Males | 44.1 | 33.8 | 22.1 | 100.0; 136 |
| Age (years) | | | | |
| 14 | 61.5 | 20.0 | | 100.0; 65 |
| 15 | 45.9 | 37.7 | | 100.0; 61 |
| 16 | 49.2 | 34.9 | | 100.0; 63 |
| 17–18 | 56.1 | 24.4 | | 100.0; 41 |

## DISCUSSION

The message promoted by AG, and appearing on the program's educational materials, proclaims that "Physical Activity is Health: Accumulate 30 minutes Per Day." In their responses, the participants referenced the educational and motivational perspectives of the program and the importance of reducing physical inactivity. The responses of "Educate for PA and Health" could be expected and are based on, in part, these youths' involvement in a program providing health information. However, these participants' direct articulation of specific physiological and physical health benefits to be derived from regular PA also demonstrate a level of incorporation, understanding, and communication about the information previously found only in adult populations[11].

The results of this case study raise important points concerning youth understanding of educational messages in PA intervention programs. A review of the intervention PA literature identified two categories of outcomes: changes in PA behaviors and changes in physical fitness measures[26]. A third important outcome deserving attention is identified by this investigation: assessment of knowledge held about PA in relation to health. The results of both the qualitative and quantitative analyses suggest that the majority of youth participants in AG who were interviewed for this study are able to articulate this program's message of health benefits from regular PA.

The 2002 "Recommendations to Increase Physical Activity in Communities" published by the Task Force on Community Preventive Services (CDC) strongly recommended a "community-wide campaign" approach to PA interventions identifying benefits in both "knowledge about exercise and physical activity and in intentions to be physically active"[27]. The results of the data presented here about AG strongly suggest a relationship between knowledge of the health benefits of regular engagement in PA and PA behavior.

The responses to the question "What are the objectives of AG?" suggest that an educational motif for an intervention can coexist with enjoyment by its participants. This has important implications for intervention programs. The ability of participants to relate PA to health regardless of the stated purposes of AG assures that it has not been perceived as doctrinaire. Instead, the educational component of AG has informed the participants' understanding of the role of PA for health in their daily lives[1]. The educational materials are made available to communities who then design a strategy appropriate for their specific context, population, resources, and needs. There are critical implications for intersecting health knowledge with changes in behavior for public health policy related to PA.

The results of this project demonstrate that spending billions of dollars on promoting PA[28] may not be necessary; what is necessary is a program that adequately combines information, constancy in its programming, and varied opportunities to match the interests of the participants and the requirements of individual communities. The results presented here showing that most of the participants understood and were able to articulate this program's educational and health-related message provide an important criterion for the development of PA intervention programs for younger populations.

## ACKNOWLEDGMENTS

This research was partially funded by the following Brazilian agencies: Fundação de Amparo à Pesquisa do Estado de São Paulo (FAPESP–Auxílio Pesquisa and Bolsa Pós-Doutorado), Coordenação de Aperfeiçoamento de Pessoal de Nível Superior (CAPES–Programa Professor Visitante Estrangeiro–PVE CAPES), and was made possible by the Bi-lateral Faculty Exchange Agreement between San Francisco State University and the University of São Paulo. This study was also made possible by the volunteer research assistance provided by eight graduate and 23 undergraduate students from the Department of Human Movement Pedagogy, of the University of São Paulo. The authors also extend their appreciation to CELAFISCS (Centro do Estudos do Laboratório de Aptidao Física de Sao Caetano do Sul) for providing access to the participants and program of Agita Galera.
